# Fire Resistance of High-Volume Fly Ash RC Slab Inclusion with Nano-Silica

**DOI:** 10.3390/ma14123311

**Published:** 2021-06-15

**Authors:** Mohamed H. Mussa, Noor Azim Mohd Radzi, Roszilah Hamid, Azrul A. Mutalib

**Affiliations:** 1 Department of Civil Engineering, Faculty of Engineering & Built Environment, Universiti Kebangsaan Malaysia, Bangi 43600, Malaysia; p100973@siswa.ukm.edu.my (N.A.M.R.); roszilah@ukm.edu.my (R.H.); azrulaam@ukm.edu.my (A.A.M.); 2 Department of Civil Engineering, College of Engineering, University of Warith Al-Anbiyaa, Karbala 56001, Iraq

**Keywords:** fire test, high volume fly ash nano-silica concrete, ISO 834, spalling and cracks, thermal conductivity

## Abstract

The study aims to investigate the fire performance of reinforced concrete (RC) slab fabricated from high volume fly ash inclusion with nano-silica (HVFANS) under ISO 834 load curve. The HVFANS concrete slab with dimensions of 1850 mm × 1700 mm × 200 mm was tested via an electrical furnace under an exposing temperature of 1100 °C for 120 min. The slab behaviour was evaluated in terms of residual compressive strength, temperature distribution along its thickness, spalling, and cracks. The results revealed that the slab was capable of maintaining 62.19% of its original compressive strength at room temperature after exposure to the above temperature. Moreover, the distribution of temperature revealed that the temperature of concrete cover and bottom reinforcement was less than 300 °C with a maximum spalling depth of 11 mm within the temperature range of 680 to 840 °C. Furthermore, the thermal conductivity index (K) of the HVFANS concrete was determined, and results indicated that thermal conductivity equalled 0.35 W/mK which is considered low, as compared with other concretes tested in current and previous studies.

## 1. Introduction

Fire represents one of the most severe conditions to which structures might be subjected as the instant the catastrophic fire of the Mont Blanc tunnel in 1999s which caused 39 deaths and serious damage to the tunnel lining structure that cost €206 to €250 million for restoration work [1,2,3,4]. In the event of a fire inside a structure, for example, a tunnel, the temperature may rise extremely fast within 30 min and reach up to 1200 °C according to temperature–time curve for road tunnels in Germany Zusätzliche Technische Vertragsbedingungen und Richtlinien für Ingenieurbauten (ZTV-ING) [5]. Therefore, providing an appropriate fire safety measure for concrete members is an important aspect of design.

Engineering structures are usually constructed from normal strength concrete (NSC) or high strength concrete (HSC) fabricated by various procedures [6,7]. Studies [8,9,10,11] revealed that the high strength concrete (HSC) lost about 40% of its room compressive strength at the intermediate temperatures range between 100 to 400 °C, while the normal strength concrete (NSC) lost approximately 20 to 30% of its strength at similar temperatures. Moreover, the laboratory tests indicated that the explosive spalling of HSC was higher than NSC due to its low permeability. This tendency means that HSC structural elements might be more susceptible to lose the concrete cover that provides thermal protection of the steel reinforcement. Consequently, it is recommended that the temperature of steel reinforcements should not exceed a range of 250 to 300 °C [12]. In this context, the HSC structures are usually protected against fire by using passive and active protection methods. Active methods, such as water sprinkler systems, are used to remove the heat from the void and cooling the structure members. In the passive protection method, the structures are usually protected by using insulating materials to restrict the flow of heat into the structure. Studies concur that adding polypropylene fibre (PPF) with a ratio between 0.5 to 3 kg/m^3^ could significantly improve the spalling resistant of HSC due to the melting of PPF between 160 to 170 °C and evaporation at 350 °C caused new pores which work as channels to relieve the internal energy of water vapour pressure [13,14,15,16,17,18]. 

Nowadays, the massive production of Portland cement resulted in significant growth in environmental pollution. Several reports stated that the manufacturing of one ton Portland cement liberated around one ton of gases to the atmosphere, mainly composed of carbon dioxide CO_2_ which contributed about 65% of global warming [19,20,21,22]. Thus, researchers suggested alternatives binders as instant fly ash (FA), slag, nanomaterial, metakaolin, etc. to decrease the reliance on Portland cement in constructions [23].

In this field, several scholars aimed to use fly ash which is a by-product of thermal electric plants as a partial replacement of Portland cement due to its beneficial effects on the concrete behaviour and low cost. Moreover, they intensively studied the effects of using FA on the thermal properties of concrete. Bentz et al. [24] reported that the increase of FA content significantly reduced the thermal conductivity of concrete, while Gifford and Ward [25] stated that the high quantities of FA slightly decreased the thermal expansion of concrete. Rashad [26,27] noted a higher relative compressive strength for the concrete containing 70% of FA after being exposed to temperatures of 400, 600, 800, and 1000 °C for 2 h, compared to the Plain Concrete (PC). Shang and Yi 2013 [28] studied the thermal properties of HSC containing 20 and 21% of fly ash with compressive strengths of 50 and 60 MPa, respectively. The results revealed that both concretes were capable of maintaining 80.4 and 81.5% of their original strengths after being exposed to 500 °C.

The main shortcoming of using high volumes of fly ash in concrete is the sharp reduction in the compressive strength at early ages as indicated by several researchers [29,30,31,32]. Therefore, nano-silica (NS) was broadly used to improve the mechanical properties of concrete containing a high volume of fly ash (HVFA), particularly at an early age, due to its pozzolanic reactivity besides the pore-filling effect. Li [33] stated that replacing 4% of NS in concrete contained 50% of FA could enhance the compressive strength by 68.57, 39.34, 18.72, and 7.58% at curing ages of 7, 28, 56, and 112 days, respectively. Ibrahim [34] replaced the HVFA concrete containing 55% of FA with colloidal NS of 2.5, 5, and 7.5% and observed that the compressive strength increased with the increase of NS content in all the investigated cases at age of 28 days.

Moreover, researchers noted that the addition of NS considerably improved the thermal stability of the HVFA concrete. Lim et al. [35] indicated that samples containing Nano silica showed lesser strength loss after being exposed to elevated temperatures at 500 °C, as compared with samples contained silica fume. Mortar containing HVFA showed higher residual strength after exposure to 700 °C, and dehydration of C–S–H produced calcium silicate which acts as a new binding material to retain residual strength [36]. Ibrahim [34] reported that high volume fly ash inclusion with nano-silica (HVFANS) concrete can be used as a fireproof material under temperatures reach up to 700 °C, whereas the HVFANS concrete was able to maintain about 94.54% of its room compressive strength after exposing to a temperature of 700 °C.

According to the above results, this study aims to test the fire performance of HVFANS concrete as a part of the structure to increase the confidence of using this type of concrete in engineering structures. Therefore, a concrete slab with dimensions of 1850 mm × 1700 mm × 200 mm was fabricated according to the mix proportions recommended by Ibrahim [34] and tested under temperatures reach up to 1100 °C via an electric furnace based on ISO 834 [37] fire load curve during the time of 120 min.

## 2. Materials and Methods

### 2.1. Materials

The Portland cement (Type I) manufactured by Tasek Cement Company (Ipoh, Perak, Malaysia) according to Malaysian standard (MS 522) [38] was used. The Fly ash was collected from Jimah Power Plant located in Malaysian Port Dickson (Negeri Sembilan, Malaysia) and categorised as class F based on British Standard (BS EN 450: 2005), which specified that the sum of silicon dioxide SiO_2_, aluminium oxide Al_2_O_3_, and ferric oxide Fe_2_O_3_ must be more than 70% [39]. The chemical component details of cement and fly ash are described in Table 1 [40].

Colloidal nano-silica (NS) type Cembinder W8 provided by AkzoNobel Company (Haman-gun, Gyeongsangnam-do, South Korea) was used to enhance the early compressive strength of concrete, thereby shortening the setting time of the cement slurry [36]. The surface area of NS particles was equal to 80 m^2^/g, with an average size of 35 nm, and a silica concentration of 50%, with a bulk density of 1050 kg/m^3^ and pH of 10. Natural river sand was used as a fine aggregate which could pass through a 4.75 mm sieve size with a bulk specific gravity of 2.53 and fineness modulus of 2.98 [41,42]. The loose and compacted bulk densities of fine aggregate were 1510.18 and 1721.83 kg/m³, respectively [43]. Crushed granite coarse aggregate provided from local sources with a maximum size of 10 mm and bulk specific gravity of 2.07 was used in the mix design. These fibres have a white colour with a melting point that ranges between 160 to 170 °C and tensile strength of 0.36 kN/mm^2^.

Superplasticiser type (Darex Super 20) manufactured by GCP Applied Technologies company (Selangor, Malaysia) was added with a dosage of 1% of cementitious materials weight to provide the required workability and improve the compressive strength of concrete via decreasing the amount of water in the mixture due to the existence of naphthalene sulphonate [44,45,46]. White-colour fibrillated polypropylene fibres (PPF), provided by Timuran Company (Selangor, Malaysia) with a dosage of 1 kg/m^3^ of concrete volume and length of 12 mm with a specific gravity of 0.9, were added to increase the ductility of concrete and reduce the surface spalling, as recommended in prior studies [13,14,15,16,17].

### 2.2. Concrete Mixture Design

The mixture proportions of plain concrete (PC) and HVFANS concrete slabs with a target compressive strength of 60 MPa were selected based on the American Concrete Institute Standard (ACI 211.4R-93) [47], as described in Table 2. The percentages of FA and NS as cement replacement equalled 52.5% and 2.5%, respectively, with a water-to-cementitious material ratio (w/c + p) of 0.29 [36].

The mixing of materials based on ASTM C192 [48] led to the formation of cement balls, mainly in the case of HVFNS concrete, since the mixture was stiff. Therefore, the sand and a portion of gravel with cement and fly ash were initially mixed. Afterward, the nano-silica and water were added. The slump test was carried out before adding the superplasticiser and maintained between 25 and 50 mm (true slump). Lastly, the rest of the gravel with PPF was added. Three concrete cubes, each with a size of 150 mm × 150 mm × 150 mm, were cast to determine the required compressive strength before casting the concrete slab. The samples were covered by a plastic membrane, to avoid moisture evaporation, and de-moulded after 48 h. The results indicated that the average value of compressive strength of the concrete was 62.7 MPa after 28 days.

### 2.3. Concrete Slab Fabrication

Two slabs were fabricated from plain concrete (PC) and HVFANS concrete, each with a size of 1850 mm × 1700 mm × 200 mm, according to the European Federation of National Associations Representing Concrete (EFNARC) [49]. During the test, the specimen dimensions were larger than the size of the furnace opening to ensure a stable position and prevent heat from escaping from the sides of the slab. Bar reinforcements with a diameter of 12 mm each and at a spacing of 100 mm × 200 mm were used. Four thermocouples manufactured by RS Components Sdn Bhd P.O (Kuala Lumpur, Malaysia) type (K), each with a diameter of 3 mm, were used and placed at depths of 30, 60, 90, and 200 mm from the bottom surface of the concrete slab. The thermocouples were installed during the casting of the slab to ensure high coupling efficiency, avoid air space, and provide full physical contact between the thermocouple and concrete.

### 2.4. Furnace Test

**Figure 1:**
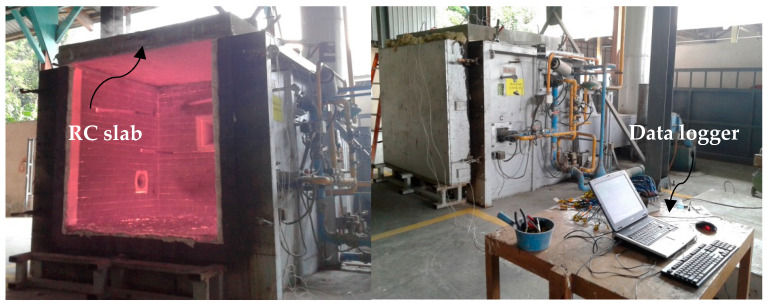
Medium-scale furnace.

The fire test was conducted by using a medium-scale furnace provided by Forest Research Institute Malaysia (FRIM) (Kepong, Selangor, Malaysia) with dimensions of 1.5 m × 1.5 m × 1.5 m at a temperature of 1100 °C, as shown in Figure 1.

**Figure 2:**
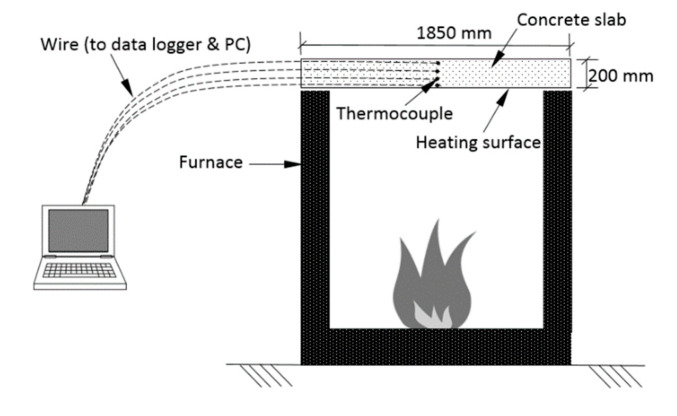
Fire test setup.

The heat within the chamber was controlled at the standard heating temperature according to the ISO 834 fire curve. The temperature was measured at 100 mm from the furnace wall to ensure compliance with the fire load curve. The opening of the furnace was wrapped by a fireclay rock wool sheet to avoid thermal loss. The concrete slab was placed horizontally at the top of the furnace opening. The test setup of the fire test included the position of the concrete slab and thermocouple wire connected to the data logger and computer, as shown in Figure 2.

**Figure 3:**
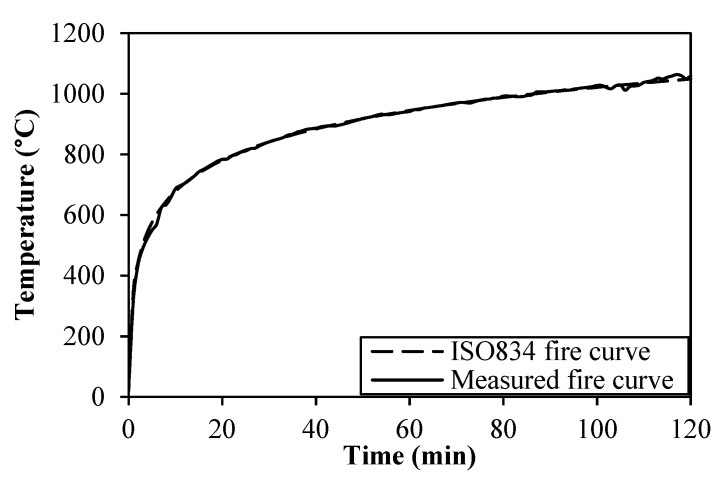
Time history of the recorded fire curves.

Based on ISO 834-1 [41], the temperature increment within the furnace is described by the following equation:

(1) $$T_{\left(t\right)}=20+345\log\left(8t+1\right)$$

where *t* is the time in min, and *T*_(*t*)_ is the gas temperature within the furnace in °C. The measured time–temperature curve in the furnace chamber, as compared with ISO 834 curve, is presented in Figure 3. A heating duration of 120 min was used during the test to simulate a real fire scenario in tunnels according to EFNARC guidelines [49]. The fire test started at 50 °C and terminated after achieving the required fire load curve. The cooling phase was monitored based on the specified fire load. The furnace was shut down after 120 min and the specimen was left into the furnace to cool down and was removed after 1 h.

### 2.5. Thermal Conductivity Test

The thermal conductivity test of PC and HVFANS concrete was carried out by using the λ-Meter EP500e tool manufactured by Lambda-Meßtechnik GmbH Dresden (Dresden, Germany) based on Malaysian Standard ISO 8302 [41]. According to the guidelines, the recommended thickness of the measured specimens should range between 10 to 200 mm to exclude the influence of heat radiation. Accordingly, the specimen size of 500 mm × 500 mm with a measured thickness of 52.93 mm and density of 2300 kg/m^3^ was used and prepared with a flat and dry surface. The thermal resistance (*R*) and thermal conductivity (*K*) are computed using the following equations:

(2) $$R=\frac{\;\;\;\Delta T\times A}{\;\;Q}$$

(3) $$K=\frac{\;d}{\;R}$$

where Δ*T* is the temperature difference, *A* is the surface area, *Q* is the total heat supplied, and *d* is the specimen thickness. The measurement was computed at a single temperature of 20 °C at a temperature difference of 15 K.

### 2.6. Coring Test

The coring tests for PC and HVFANS concrete slabs were conducted before and after the fire test according to ASTM C42 guidelines which recommended that the ratio of specimen length to its diameter (L/D) should range between 1.9 to 2.1 [50]. Therefore, the cylindrical drilled cores specimens with dimensions of (∅50 mm × 100 mm were tested. A total of 12 cores were drilled, whereas 4 cores were used to evaluate the percentage of free moisture content and the rest were utilised to determine the residual compressive strength and density of both slabs.

## 3. Results and Discussion

### 3.1. Results of Thermal Conductivity Test

The thermal conductivity of PC and HVFANS concrete was determined. The results demonstrated that the HVFANS concrete had a lower thermal conductivity equal to 0.35 W/mK, as compared with PC, which recorded 0.8 W/mK at similar test circumstances. This behaviour might be attributed to the existence of high-volume fly ash, whereas Bentz [24] recorded a significant reduction in the thermal conductivity, by 19%, for HVFA concrete that contained 75% fly ash, as compared with conventional concrete. Moreover, using nano-silica in concrete mixture decreased the pore size and total porosity of the concrete due to the large surface area production that may speed up the rate of the pozzolanic reaction and cause a lower conductivity [36].

### 3.2. Furnace Test

#### 3.2.1. Temperature Distribution along the Slab Thickness

**Figure 4:**
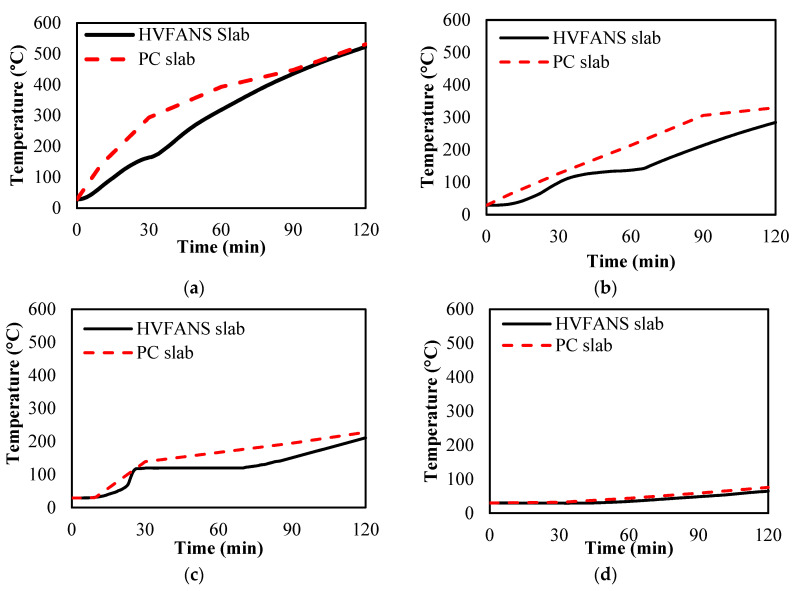
Temperature distribution of PC and HVFANS slabs at depths: (**a**) 30 mm; (**b**) 60 mm; (**c**) 90 mm; (**d**) 200 mm, from the exposed surface.

During the fire test, the thermal distribution of PC and HVFANS concrete slabs was recorded via four thermocouples located at different depths from the heated surface, as shown in Figure 4. At depth 30 mm, the HVFANS slab showed lower thermal distribution at all the time intervals, as compared to the PC slab, particularly after 10 to 30 min, by −51% and −44.08%, respectively. These differences were reduced with the increase of time and recorded −1.56% after 2 h of fire with a maximum temperature of 531 °C. At the depth of 60 mm, once again the HVFANS slab showed lower temperature magnitudes in all the time periods with a maximum temperature of 285 °C after 2 h. This behaviour may be attributed to the low thermal conductivity of HVFANS concrete, as compared to PC. At the depth of 90 mm, the recorded temperature of the HVFANS slab was lower than the PC slab by −13.88% after 30 min. A constant temperature equal to 120 °C was noted during 30 to 70 min for the HVFANS slab, as a result of the evaporation of free water and chemically bonded water of the calcium silicate hydrate (C–S–H) from the concrete slab [51]. The temperature started to increase slightly after 73 min until it reached up to 211 °C within 120 min. Finally, at the depth of 200 mm, the temperatures were considerably reduced for both slabs; nevertheless, the HVFANS slab still showed a lower thermal distribution at all time intervals with a maximum difference of −21.14% after 1 h and the highest temperature of 76 °C after 2 h, as shown in Figure 4d.

Further comparison was conducted with previous slabs tested by other scholars under similar temperature magnitudes, reaching up to 1100 °C, which particularly occurred in concrete tunnels [52,53]. The results proved that HVFANS concrete slab had better performance and recorded lower temperatures particularly at a depth of 60 mm which is considered the standard depth of concrete cover and reinforcement location for these structures, as shown in Table 3.

#### 3.2.2. Concrete Spalling

**Figure 5:**
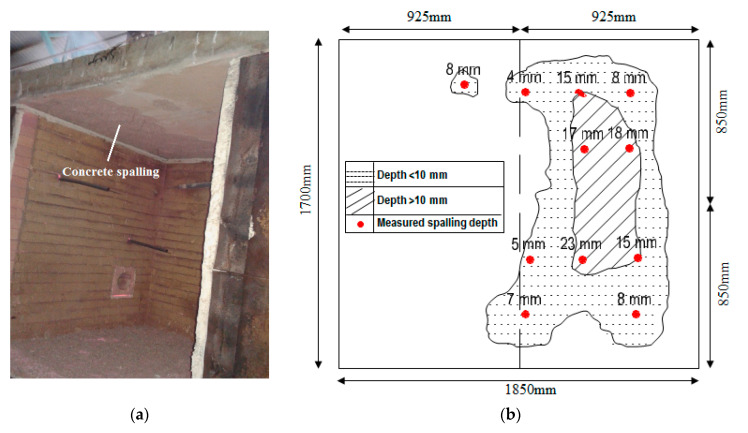
Concrete spalling: (**a**) visual appearance and (**b**) coverage area.

**Figure 6:**
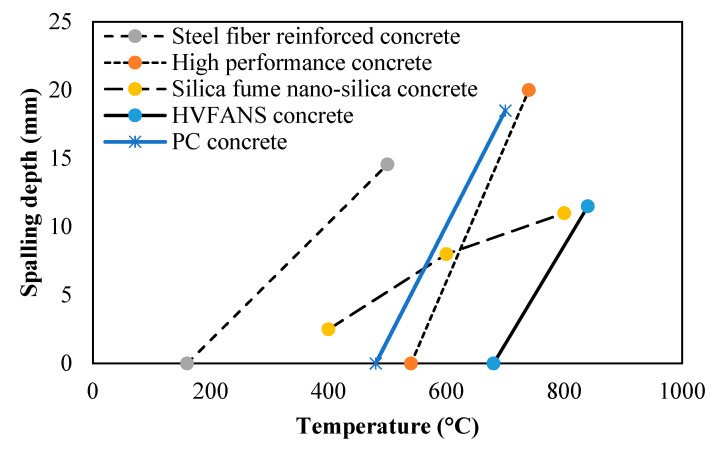
Concrete spalling depth at different temperatures [53,54,56].

The concrete spalling effect was carefully observed and recorded during the furnace test for both concrete slabs. The results showed a spalling location on the heated surface of the HVFANS concrete slab with a maximum depth of 11 mm and area of 34.3% within the temperature range of 680 to 840 °C, while the PC slab recorded the highest spalling depth of 19 mm within a lower temperature range of 480 to 700 °C, as shown in Figure 5 and Figure 6. Moreover, the outcomes were compared with previous studies using slabs fabricated from different materials and were tested by using an ISO 834 fire curve; once again, the HVFANS concrete slab showed a superior behaviour, as compared with these slabs [54,55,56,57]. No concrete spalling occurred in both concrete slabs during the cooling period. Ali et al. [58] indicated three types for concrete spalling, namely, minor, major, and severe, according to the depth of spalling. Accordingly, the spalling of HVFANS concrete could be considered minor since the fire did not reach the reinforcement depth. High-strength concrete usually contains vapour pressure in its pores and also suffers from large thermal stress. In this study, using PPF and small-size aggregate (10 mm) significantly relieved the thermal expansion and stresses induced in concrete, as reported by Maraveas and Vrakas [59].

#### 3.2.3. Moisture Content

The moisture content of both concrete slabs was recorded before and after the fire test. The average records of the cores for PC and HVFANS concrete before the fire were 4.95 and 5.97%, respectively. The moisture content was reduced after firing for both concrete slabs and recorded 4.10 and 5.40%. The water was vapoured out from visible cracks distributed along the width of the unexposed concrete slab surface as well as along the installation holes of the thermocouples and reinforcement within the time range between 20 to 100 min. Similar phenomena were observed by previous scholars [56,60,61,62]. Zeiml et al. [63] indicated that the cracks occurred because of the tensile thermal stress and non-uniform temperature distribution. Using nano-silica reduced the crack formation on the slab surface [36].

#### 3.2.4. Residual Compressive Strength and Density

The average compressive strength of two cylindrical coring samples was recorded before and after the firing of PC and HVFANS concrete slabs. The results revealed that the compressive strength of both slabs was decreased after exposure to elevated temperatures reaching up to 1100 °C. However, the HVFANS slab showed superior fire resistance and recorded the lowest reduction, by −37.81%, as compared with the PC slab which recorded a reduction of −63.42%, as shown in Table 4.

Moreover, the reduction and relative compressive strength (RCS) was computed for both concrete slabs by the following equations:

(4) $$Reduction\;\left(\%\right)=\frac{{\left(f_{c\;}\right)}_{after\;fire}-{\left(f_{c\;}\right)}_{before\;fire}}{{\left(f_{c\;}\right)}_{before\;fire}}\times 100$$

(5) RCS=100−Reduction(%)

The results proved that the HVFANS slab had the highest RCS by 62.19%, as compared with PC which recorded only 36.58%. Ibrahim et al. [36] conducted an X-ray diffractometry (XRD) test and stated that the high residual strength at temperatures above 700 °C for the concrete containing similar proportions of fly ash and nano-silica, shown in Table 1, may be attributed to the formation of new silicate compounds from the reaction of the fly ash and nano-silica which caused a significant reduction in calcium silicate. Moreover, Ibrahim et al. [36] observed superior performance for these in terms of the pore size distribution and recorded a higher decrease, as compared to control samples, because of the great stability of the calcium silicate hydrate, as indicated in the scanning electron microscope (SEM) test.

From the above core samples, the density of PC and HVFANS concrete slabs were determined before and after the fire test according to ASTM C138 [64]. The results proved that the density of both slabs was reduced at high temperatures; however, the HVFANS slab showed better performance and recorded a reduction of −7.93%, as shown in Table 5.

#### 3.2.5. Steel Bearing Capacity

**Figure 7:**
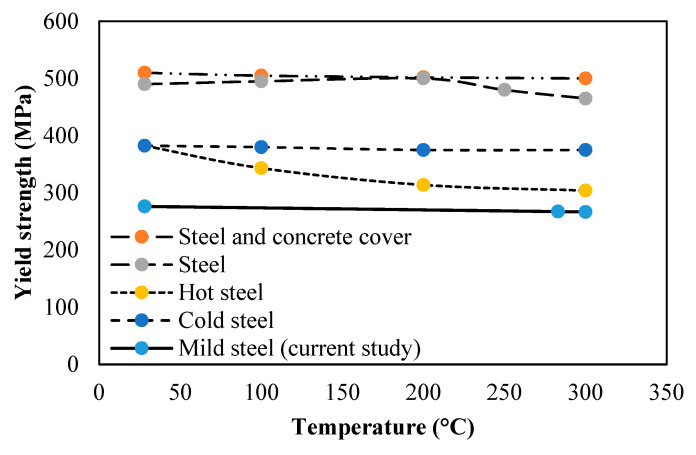
Yield strength of reinforcement at different temperatures [65,66].

A rebar tensile test was performed to determine the yield strength of steel reinforcement before and after the fire test. The yield strength of mild steel reinforcement used in the HVFANS slab almost remained constant, with a value of 276.29 MPa between temperatures of 28 to 283 °C, as shown in Figure 7. However, it was slightly decreased by 3.4% and recorded 266.68 MPa at a temperature of 300 °C.

The mechanical properties of the steel reinforcement deteriorated when subjected to temperatures exceeding 300 °C [59]. The results proved that the cover of 60 mm used in the HVFANS concrete slab was capable to withstand elevated temperatures reaching up to 1100 °C, while this cover was not sufficient to maintain the temperature of rebar less than 300 °C, and the temperature was able to reach into the slab reinforcement and caused severe damage. Unluoglu et al. [65] and Naus [66] observed similar deterioration in the reinforcement within various percentages that ranged between 2.0 and 20.5% and stated that a thicker concrete cover could reduce the temperature of reinforcement and delay its failure which mainly depended on the peak temperature and fire duration.

## 4. Conclusions

The results can be summarised as follows:

1. Visual inspection showed that minor spalling occurred in the concrete slab with a maximum depth of 11 mm at a duration between 10 to 30 min within a temperature range of 680 to 840 °C while severe spalling occurred in the PC slab with a depth of 19 mm within temperatures range of 480 to 700 °C;
2. The coring test indicated that the residual compressive strength of the HVFANS concrete slab was reduced by −37.81% from its original compressive strength under 1100 °C, while the strength of the PC slab was reduced by −63.42% under similar circumstances;
3. The temperature distribution along slab thickness indicated that the HVFANS concrete had superior performance and recorded lower temperatures than the PC slab under all the investigated depths;
4. The yield strength of mild steel reinforcement into the HVFANS concrete slab almost remained constant with a value of 276.29 MPa between temperatures of 28 to 285 °C, while the strength of similar reinforcement into the PC slab was reduced to 216.29 MPa under identical conditions;
5. The thermal conductivity test results proved that HVFANS concrete had excellent fire resistance with a maximum thermal conductivity value (*K*) of 0.35 W/mK, which is considered lower than other concrete types such as NSC and HVFA concrete types.

According to the above results, the HVFANS concrete can provide superior fire protection, as compared to plain concrete, for the structural members exposed to massive temperatures reaching up to 1100 °C. Therefore, future studies are recommended to present further information about this type of concrete.

## Figures and Tables

**Table 1:** Chemical detail of cement and fly ash materials (Adapted from [40]).

Items	Clinker (%)	Cement (%)	Fly Ash (%)
Silicon dioxide, SiO_2_	21.66	21.28	44.16
Aluminium oxide, Al_2_O_3_	5.8	5.6	24.6
Ferric oxide, Fe_2_O_3_	3.68	3.36	12.5
Calcium oxide, CaO	65.19	64.64	5.34
Magnesium oxide, MgO	2.86	2.06	2.5
Titanium dioxide, TiO_2_	-	-	4.1
Potassium oxide, K_2_O	-	-	1.5
Sulphur trioxide, SO_3_	0.2	2.14	0.3
Total alkalis	0.07	0.05	-
Insoluble residue	0.1	0.22	-
Loss on ignition, LOI	0.27	0.64	5

**Table 2:** PC (Plain Concrete) and HVFANS (high volume fly ash inclusion with nano-silica) concrete mixing proportions (kg/m^3^).

Concrete Type	Cement	Fly Ash	Nano-Silica	Water	Gravel	Sand	PPF (Polypropylene Fibres)	Superplasticiser
PC	538.13	-	-	181.16	959.32	717.39	1.0	5.38
HVFANS	236.25	275.63	26.25	165.82	959.32	619.34	1.0	5.38

**Table 3:** Comparison of temperature recorded at depth 60 mm after 2 h heating in previous studies.

Researcher	Materials	Temperature (°C)
Current study	HVFANS concrete slab	285
Dorgarten et al. [53]	Concrete made by HOCHTIEF company	370
Caner and Böncü [52]	High strength concrete with micro polypropylene fibres	290 and 305

**Table 4:** Compressive strength ($f_{c}$ ) of PC and HVFANS slabs before and after firing.

Slab Type	Condition	Core Reference	$f_{c}\;(\mathrm{MPa})$	Average (MPa)	Reduction (%)	RCS (%)
HVFANS	Before fire	C1	74.62	74.39	−37.81	62.19
		C2	74.16			
	After fire	C3	46.91	46.26		
		C4	45.61			
PC	Before fire	C1	76.83	77.04	−63.42	36.58
		C2	77.24			
	After fire	C3	27.47	28.18		
		C4	28.88			

**Table 5:** Density (ρ ) (kg/m^3^) of PC and HVFANS slabs before and after firing.

Slab Type	Condition	Core Reference	Volume (m^3^)	Weight (kg)	ρ	Average
HVFANS	Before fire	C1	0.00019	0.440	2316	2308
		C2	0.00019	0.437	2300	
	After fire	C3	0.00018	0.386	2144	2125
		C4	0.00017	0.379	2106	
PC	Before fire	C1	0.00018	0.439	2439	2446
		C2	0.00019	0.466	2453	
	After fire	C3	0.00018	0.37	2056	2078
		C4	0.00018	0.378	2100	

## Data Availability

All the data is available within the manuscript.
